# Comparison of multilevel modeling and the family-based association test for identifying genetic variants associated with systolic and diastolic blood pressure using Genetic Analysis Workshop 18 simulated data

**DOI:** 10.1186/1753-6561-8-S1-S30

**Published:** 2014-06-17

**Authors:** Jian Wang, Robert Yu, Sanjay Shete

**Affiliations:** 1 Department of Biostatistics, The University of Texas MD Anderson Cancer Center, Houston, TX 77030, USA; 2 Department of Epidemiology, The University of Texas MD Anderson Cancer Center, Houston, TX 77030, USA

## Abstract

Identifying genetic variants associated with complex diseases is an important task in genetic research. Although association studies based on unrelated individuals (ie, case-control genome-wide association studies) have successfully identified common single-nucleotide polymorphisms for many complex diseases, these studies are not so likely to identify rare genetic variants. In contrast, family-based association studies are particularly useful for identifying rare-variant associations. Recently, there has been some interest in employing multilevel models in family-based genetic association studies. However, the performance of such models in these studies, especially for longitudinal family-based sequence data, has not been fully investigated. Therefore, in this study, we investigated the performance of the multilevel model in the family-based genetic association analysis and compared it with the conventional family-based association test, by examining the powers and type I error rates of the 2 approaches using 3 data sets from the Genetic Analysis Workshop 18 simulated data: genome-wide association single-nucleotide polymorphism data, sequence data, and rare-variants-only data. Compared with the univariate family-based association test, the multilevel model had slightly higher power to identify most of the causal genetic variants using the genome-wide association single-nucleotide polymorphism data and sequence data. However, both approaches had low power to identify most of the causal single-nucleotide polymorphisms, especially those among the relatively rare genetic variants. Therefore, we suggest a unified method that combines both approaches and incorporates collapsing strategy, which may be more powerful than either approach alone for studying genetic associations using family-based data.

## Background

Identifying genetic variants associated with complex diseases is an important task in genetic studies, including genome-wide association (GWA) studies and whole-genome sequencing studies. Although association studies based on unrelated individuals (ie, case-control GWA studies) have successfully identified common single-nucleotide polymorphisms (SNPs) in many complex diseases, these studies are not so likely to identify rare genetic variants. In contrast, family-based association studies have the ability to identify rare variants. Moreover, a family-based study design can avoid the problem of population stratification, tend to be more homogeneous regarding early exposure to environmental factors, and test both linkage and association [1,2].

Multilevel models are statistical models with parameters that vary at more than 1 level [3] and have been widely used in social, behavioral, business, marketing, and economic studies in which the empirical data exhibit a hierarchical structure. Recently, there has been some interest in employing multilevel models in family-based genetic association studies [2,4]. However, the performance of multilevel model analysis in family-based genetic association studies, especially for longitudinal family-based sequence data, has not been fully investigated. Consequently, in this study, our aim was to examine the performance of multilevel model analysis in family-based association study, compared with that of the more commonly used family-based association test (FBAT) [5,6]. We investigated the powers and type I error probabilities of both approaches using simulated GWA, sequence, and rare-variants-only data provided by Genetic Analysis Workshop 18 (GAW18), with knowledge of the simulation model.

## Methods

### Simulation data

For GAW18, 200 replicates of simulated longitudinal phenotype data were available that had been generated utilizing the real pedigree structures, the imputed sequence data, and distributions of phenotypes [7]. The available phenotypes were systolic and diastolic blood pressure (SBP and DBP), hypertension, and smoking status, which were simulated for 849 individuals at 3 time points, with no missing values. The available covariates were age, sex, and use of antihypertensive medications. We investigated the SBP and DBP measures. Because they had been adjusted according to medication use in the simulation, we did not perform further adjustments in our analyses.

To investigate the powers and type I error rates of both the multilevel model and the FBAT approach, we selected causal and noncausal genetic variants, respectively, using data from GWA and sequence studies. Based on the provided answers, we found that there were 1457 causal genetic variants across all available chromosomes, of which 1020 variants were causal for SBP and 1215 variants were causal for DBP on the basis of the sequence data. Among these causal genetic variants, 149 were available in the GWA SNP data set, of which 105 SNPs were causal for SBP and 117 SNPs were causal for DBP. We also analyzed the relatively rare variants (minor allele frequency [MAF] <0.05) separately. We found that among all 1457 causal genetic variants, 1019 variants were relatively rare; of those, 722 were causal for SBP and 844 were causal for DBP. To assess type I error rates, we selected the noncausal SNPs that were not in linkage disequilibrium (LD) with any causal variants (*r^2 ^*<0.02 for sequence data and *r^2 ^*<0.01 and MAF >0.05 for GWA data) to ensure no indirect associations. To assess rare variants using sequence data, we selected noncausal variants with a MAF of <0.05. We assumed an additive genetic model for all variants in our analyses. We performed the multilevel model and FBAT analyses on a single variant at a time.

### Multilevel model

Intraclass correlation refers to correlation among observations within a higher-level unit (eg, family). In general, the statistical significance of the intraclass correlation coefficient (ICC) is tested to evaluate the degree of this correlation and, therefore, is used to assess whether a multilevel model is necessary in the study of a hierarchical data set [3]. In our study, on the basis of the estimated ICCs in the simulated replicates, we found that the data using sibships as the higher-level unit had much higher ICCs than that using families as the higher-level unit (average ICC, 35% for sibships vs. 3% for families). Therefore, we considered sibships in the multilevel model analysis as the higher-level unit, which resulted in 741 individuals within 310 sibships. Furthermore, we used a likelihood ratio test to compare the 2-level longitudinal data (individuals and 3 longitudinal data points for each individual) with the 3-level longitudinal data that included sibships (sibships, individuals, and 3 longitudinal data points for each individual) and found that the 3-level model was statistically a better fit. Additional likelihood ratio tests were performed to determine the inclusion or exclusion of other covariates. We found that age and sex were statistically significant and therefore included them in the model. We also performed principal component analysis to test for population stratification with the use of EIGENSTRAT 3.0 [8]. We used approximately 10,000 GWA SNPs that were not in LD with any of the 149 causal SNPs (*r^2 ^*<0.02). We included the top 10 largest principal components (PCs) as covariates in our analyses. The final, 3-level model we obtained was *Y_ijk _= β_0 _+ β_1_ × SNP_ij _+ β_2_ × SEX_ij _+ β_3_ × AGE_ijk _+ α_1_ × PC_1ij _+...+ α_10_ × PC_10ij _+ µ_i _+ τ_ij _+ e_ijk_*, where *Y_ijk _*is the SBP or DBP value at time point *k *= 1, 2, or 3 for individual *j *= 1, ..., 741 within sibship *i *= 1, ..., 310. *β_u_, u *= 0,..., 3, and *α_v_, v *= 1,..., 10, are the coefficients (fixed effects) of the intercept and slopes of different covariates, and *µ_i_, τ_ij_*, and *e_ijk _*are the error terms (random effects) between sibships, between individuals within sibships, and between time points within individuals, respectively. The multilevel model analyses were conducted using R package lme4 [9].

### FBAT

The FBATs for all the causal and noncausal genetic variants were conducted using software package FBAT 2.0.4 [5,6]. The FBAT software implements a broad class of FBATs based on an extension of the transmission disequilibrium test approach, in which alleles transmitted to affected offspring are compared with the expected alleles among offspring [5]. We employed the conventional univariate FBAT approach (using the first time point only), which uses only the within-family information to investigate the phenotype-marker association based on the single-marker analysis. As in the multilevel model analysis, 741 individuals were included in the FBAT analyses. We also conducted FBAT analysis using all 849 individuals and obtained very similar results (data not shown). We analyzed SBP and DBP phenotypes simulated for only the first time point when applying the FBAT approach. For the purpose of comparison, we included age, sex, and the top 10 PCs as covariates in the analysis, that is, we assessed residuals of SBP and DBP by accounting for all covariates and employed the residuals as the phenotypes of interest in the analyses. We further used the standard offsets minimizing the variance of the test statistics that were internally calculated by the FBAT.

## Results

We investigated powers and type I error rates for the multilevel model and FBAT approach using 3 data sets: (a) GWA SNP data, in which most of the SNPs are relatively common (MAF >0.05); (b) sequence data, which include rare and common genetic variants; and (c) rare sequence data, which include only rare genetic variants (MAF <0.05). All the results were based on the 200 simulated replicates.

### Type I error rates

All the results were based on a nominal significance level of 0.05. We did not perform multiple testing corrections owing to the small number of replicates (200). For the GWA SNP data, we employed 246 noncausal SNPs with MAF >0.05 that were not in LD (*r^2 ^*<0.01) with any of the 149 causal SNPs. When using SBP as the phenotype of interest, we found that of the 246 noncausal SNPs, 76 SNPs in the multilevel model analysis, and 77 SNPs in the FBAT approach had inflated type I error rates (ie, more than 10 replicates [out of 200 replicates] with *p *values <0.05). The average type I error rates across all noncausal SNPs were 0.05 (SE = 0.05) for the multilevel model and 0.047 (SE = 0.035) for the FBAT approach. Therefore, the type I error rates for both approaches are comparable for the GWA SNP data (ie, common variants). For the sequence data (rare and common variants), we employed 13,440 noncausal genetic variants that were not in LD (*r^2 ^*<0.02) with any of the 1457 causal variants. Using SBP as the phenotype of interest, of the 13,440 noncausal variants, we observed 4357 variants with inflated type I error rates using multilevel model analysis and 3958 variants with inflated type I error rates using the FBAT approach. The average type I error rates across all noncausal variants were 0.06 (SE = 0.074) for the multilevel model and 0.046 (SE = 0.041) for the FBAT approach. From the noncausal genetic variants in the sequence data set, we selected 218 variants with MAF <0.05 (ie, rare variants). Using SBP as the phenotype of interest, of these 218 variants, we observed 68 variants with inflated type I error rates using multilevel model analysis, with an average type I error rate of 0.058 (SE = 0.075). The proportion of variants with inflated type I error rates and the average type I error rate was very similar to that in the studies of the first 2 data sets. However, for the FBAT approach, only 2 variants had inflated type I error rates; the average type I error rate using the FBAT approach to analyze rare variants was 0.007 (SE = 0.013). Because the type I error rates of the 2 approaches for the study of rare-variants-only data are not comparable, we conducted the power calculations for both approaches by adjusting for their type I errors. Specifically, using *p *values from the 200 replicates for noncausal variants (null distribution of *p *values), we identified thresholds for the multilevel model and FBAT approach that correspond to the controlled type I error rates and then computed powers based on these thresholds. We also investigated type I error rates using DBP as the phenotype of interest and obtained similar results (data not shown).

### Power comparisons

**Figure 1:**
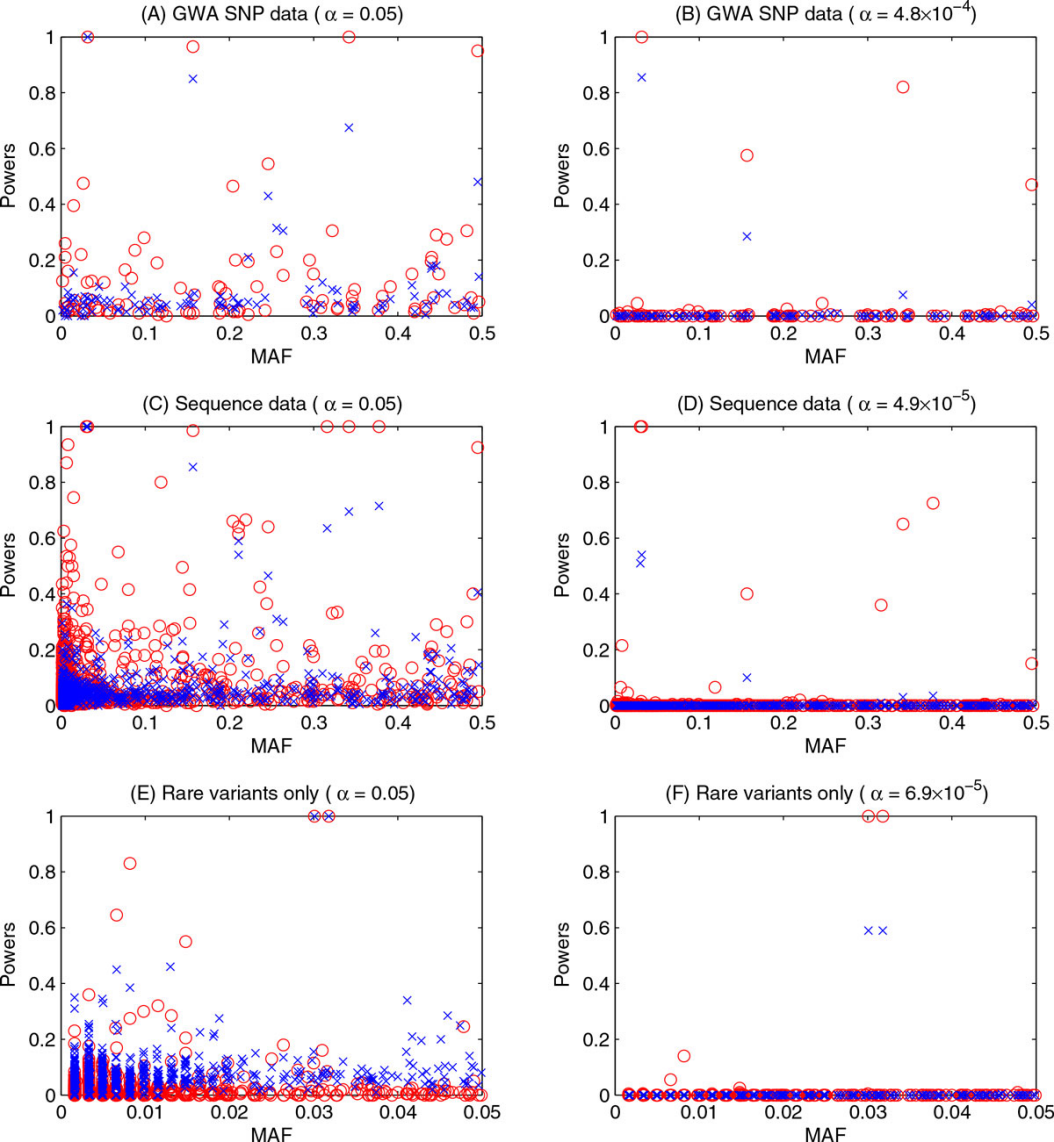
**Powers of the multilevel model and the FBAT (the first time point only) using DBP**. A and B, results obtained using GWA SNP data at 0.05 and Bonferroni-corrected significance levels, respectively; C and D, results obtained using sequence data at 0.05 and Bonferroni-corrected significance levels, respectively; E and F, results obtained using rare-variants-only data at 0.05 and Bonferroni-corrected significance levels, respectively. *FBAT*, family-based association test; *MAF*, minor allele frequency; *MM*, multilevel model. *Red circles*, results from MM; *blue × marks*, results from FBAT.

**Figure 2:**
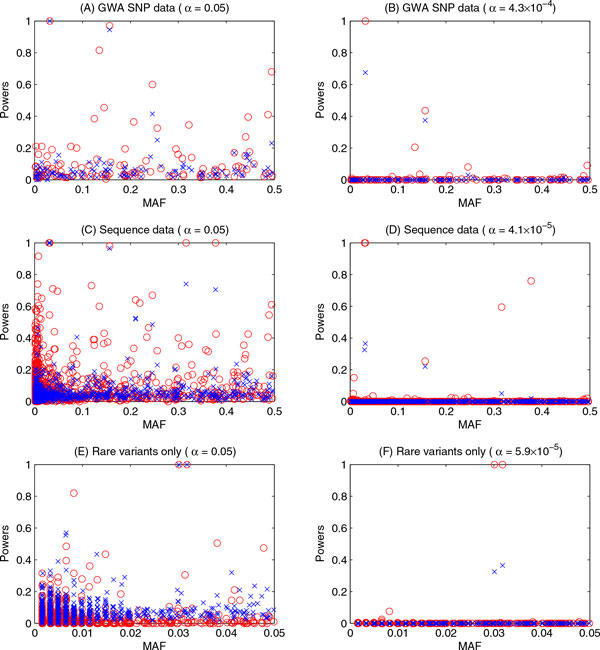
**Powers of the multilevel model and the FBAT (the first time point only) using SBP**. A and B, results obtained using GWA SNP data at 0.05 and Bonferroni-corrected significance levels, respectively; C and D, results obtained using sequence data at 0.05 and Bonferroni-corrected significance levels, respectively; E and F, results obtained using rare-variants-only data at 0.05 and Bonferroni-corrected significance levels, respectively. *FBAT*, family-based association test; *MAF*, minor allele frequency; *MM*, multilevel model. *Red circles*, results from MM; *blue × marks*, results from FBAT.

The power comparison results of the multilevel model and single-time-point FBAT approach as a function of MAFs of genetic variants are shown in Figures 1 and 2 for SBP and DBP, respectively. Both figures show the powers of 2 approaches for each of the 3 data sets with and without Bonferroni corrections. The Bonferroni-corrected significance levels differed between phenotypes and between data sets because the number of causal variants differed between those scenarios. For example, in the GWA SNP data, 105 SNPs were causal for SBP and 117 SNPs were causal for DBP; therefore, the Bonferroni-corrected significance levels were 0.05/105, or 4.8 × 10^−4^, for SBP (Figure 1B) and 0.05/117, or 4.3 × 10^−4^, for DBP (Figure 2B).

From the power results for SBP, we can observe that for most of the causal genetic variants, the multilevel model analysis had powers relatively higher than or similar to those of FBAT when using the GWA SNP data set (see Figure 1A) and the sequence data set (see Figure 1C). When using the rare-variants-only data set (see Figure 1E), both approaches had very little power for identifying almost all the causal variants (less than 20% at the 0.05 significance level). When the Bonferroni-corrected significance levels were used (see Figure 1B, D, and F), both approaches had almost no power to identify any causal variants. Moreover, the MAFs of the variants did not substantially affect the power in either approach. Meanwhile, the power results for DBP were very similar to those for SBP in all data sets (see Figure 2).

We also investigated the causal variants with power of at least 20% for both the multilevel model and FBAT approach (data not shown). Most of the causal genetic variants were removed from this set because the powers using either approach were less than 20%. However, interestingly, for the rest of the causal variants, multilevel model analysis had higher power than the FBAT approach for almost all of them.

We further investigated the relationships between power, effect size (unit = mm Hg), and MAF using the GWA SNP data set with SBP as the phenotype of interest. We found that variants with very high effect sizes could be identified with high power for both approaches; for example, rs11711953 had an effect size of −9.9107, and both approaches had powers of almost 100% to identify this variant at a 0.05 significance level; however, when the Bonferroni correction was applied, the FBAT's power decreased dramatically (85.5%), while the multilevel model's power remained at 100%. We also observed that both approaches had relatively low power to identify causal variants with high effect sizes but low MAFs and relatively high power to identify causal variants with low effect sizes but high MAFs, as has been shown in our previous study [10]. For example, for SNP rs11465293, which had an effect size of −1.1227 for SBP and a MAF of 0.0148, the powers were only 39.5% for the multilevel model and 15.5% for FBAT, whereas for variant rs1131356, which had an effect size of 1.0007 for SBP and MAF of 0.4947, the powers were 95% and 48%, respectively.

## Discussion

It is natural to consider the use of multilevel modeling for family studies because children are nested within families [11-14]. Recently, some studies have applied the multilevel model (or mixed model) to account for the hierarchical structure of families, using either families or sibships as the higher levels in the study; however, these studies did not involve longitudinal family-based data [2,15,16]. Luan et al [4] employed a 3-level model to evaluate the association between candidate genes and weight and body mass index based on Framingham longitudinal family data, using families as the highest level in the analysis. However, the performance of the multilevel model approach in identifying sequence-based genetic markers associated with complex diseases using family-based longitudinal data has not been fully investigated. In this study, using the simulated data available in GAW18, we investigated the performance of the multilevel model and compared its performance with that of the FBAT approach by examining the powers and type I error probabilities of both approaches. We did not include any longitudinal interaction term, such as interaction of a genetic variant with age, in our multilevel models because of the limited power to assess such interactive effects in this study. Notably, we tested SBP and DBP phenotypes for only the first time point in the FBAT analyses. A more commensurate comparison would be afforded by using a longitudinal test such as FBAT-GEE or FBAT-LC. Another alternative approach could use averaging measures over 3 different time points for use in the FBAT approach; however, we did not use this averaging approach in our analyses because of the missing values in the data.

We considered using 3 different data sets, including GWA SNP data, sequence genome data, and rare-variants-only data. We observed that the multilevel model had consistent type I error results for all 3 data sets; in all the scenarios, approximately 30% of the noncausal genetic variants had inflated type I error rates, and the average type I error rates across all noncausal genetic variants were controlled at a 0.05 significance level. However, the FBAT approach had different patterns of type I error rates for the different data sets. The type I error rates of the FBAT for GWA SNP data and sequence data were very similar and comparable to those of the multilevel model (~30% were inflated), but the type I error rates of FBAT for rare variants only were quite conservative. Therefore, for the purpose of power comparisons, we calculated the powers of the multilevel model and the FBAT approach by adjusting for their type I error rates when studying the rare variants only. The multilevel model had relatively higher power than FBAT for most of the causal genetic variants in the GWA SNP data and sequence data. However, both approaches had poor power to identify most of the causal variants, even those with large effect sizes, especially the relatively rare variants.

For future studies, we recommend a unified method combining both the multilevel and FBAT approaches, which may be more powerful than either approach alone for genetic association studies using family-based data. For the rare variants analysis, we can also employ the most commonly used strategy of collapsing methods, which combines the effects of multiple rare variants using a weighted linear combination and treats the combined variable as one variant in the analysis. This strategy has been used in family-based association studies, for instance, in the FBAT-Rare method, which linearly combines rare variants in a single gene [17]. We would consider incorporating the collapsing strategy into a multilevel model and further combining the multilevel model and FBAT-Rare approaches into a unified method for genetic association studies using family-based data.

## Competing interests

The authors declare that they have no competing interests.

## Authors' contributions

JW and SS conceived and designed the overall study, JW conducted statistical analyses and drafted the manuscript, and RY conducted data preparation and management. All authors read and approved the final manuscript.
